# Expectation management in AI: A framework for understanding stakeholder trust and acceptance of artificial intelligence systems

**DOI:** 10.1016/j.heliyon.2024.e28562

**Published:** 2024-03-25

**Authors:** Marjorie Kinney, Maria Anastasiadou, Mijail Naranjo-Zolotov, Vitor Santos

**Affiliations:** NOVA Information Management School (NOVA IMS), Universidade NOVA de Lisboa, Campus de Campolide, 1070-312, Lisboa, Portugal

**Keywords:** Expectation management, Artificial intelligence, Machine learning, Trustworthy AI, Explainable AI, AI development process, AI in education, AI in health

## Abstract

As artificial intelligence systems gain traction, their trustworthiness becomes paramount to harness their benefits and mitigate risks. This study underscores the pressing need for an expectation management framework to align stakeholder anticipations before any system-related activities, such as data collection, modeling, or implementation. To this end, we introduce a comprehensive framework tailored to capture end-user expectations specifically for trustworthy artificial intelligence systems. To ensure its relevance and robustness, we validated the framework via semi-structured interviews, encompassing questions rooted in the framework's constructs and principles. These interviews engaged fourteen diverse end users across the healthcare and education sectors, including physicians, teachers, and students. Through a meticulous qualitative analysis of the interview transcripts, we unearthed pivotal themes and discerned varying perspectives among the interviewee groups. Ultimately, our framework stands as a pivotal tool, paving the way for in-depth discussions about user expectations, illuminating the significance of various system attributes, and spotlighting potential challenges that might jeopardize the system's efficacy.

## 1 Introduction

Expectations about the impact of artificial intelligence (AI) systems use vary across stakeholders and sectors, with both positive and negative outcomes anticipated. While it is expected that the use of these systems can improve efficiency, enhance decision-making capabilities, and lead to improvements in the quality of life of citizens [1], in the implementation of these systems, current inequities are exacerbated, and privacy and safety issues are abundant [2]. On the one hand, the trustworthiness of these systems is key to increasing the likelihood of AI adoption, ensuring positive benefits are maximized while the potential for harm is reduced [[2], [3], [4]]. On the other hand, a mismatch between user expectations and user experience can lead to dissatisfaction with and lower adoption rates of AI systems [5,6]. Various approaches have been proposed to try to increase the reliability and trustworthiness of AI systems across multiple stakeholders to boost acceptance and adoption [[7], [8], [9]].

One contributing factor to the mismatch between individuals’ expectations and the capabilities of AI systems is the portrayal of AI in the media. Brennen et al. (2022) [10] demonstrate how media outlets create an expectation of a pseudo-artificial general intelligence, which is seen as a set of technologies capable of solving almost any problem. This can lead to extremely high expectations of AI systems in end users. They warn that not only does this overestimate the capacity of AI, but this expectation also downplays the true costs and risks of integrating AI across all sectors.

Even informed users can vary in their expectations. For example, if two stakeholders expect that data used to train the models will undergo preprocessing, they may vary in the way they think missing values are treated, one expecting instances with missing data points to be eliminated in preprocessing, while another may expect that those missing values are replaced. The treatment of missing values is likely to impact the overall functioning of the system. These different expectations, which can happen at any stage, will contribute to varied acceptance rates. Kocielnik et al. (2019) [11] demonstrate a similar issue in their study of an AI-based scheduling assistant. In the study, the authors used a scheduling assistant to test user acceptance in two scenarios: both having identical accuracy but with different false positive rates (more non-meeting requests detected in the high recall version) and false negative rates (more meetings missed in the high precision version). The high recall version led to significantly higher perceptions of accuracy and significantly higher acceptance than the high precision version, even though the accuracy rates were identical.

Defining expectations of AI systems is a challenging and difficult task, as those expectations vary widely between individuals and between applications [12]. Although literature emphasizes the need for an expectation management framework to help balance stakeholder expectations and increase trust and acceptance of the AI systems [2], recent studies have not yet provided or evaluated such a framework able to cover the trustworthiness and acceptance dimensions. For instance, Langer et al. (2021) [13] focus only on the classes of stakeholders and their requirements for explainable AI, not considering the broader range or principles that lead to trustworthiness. Chen et al. (2020) [[7], [8], [9]] proposed and evaluated a model that sheds light on the relevant factors of AI systems user experience in electronic government. However, this model has limited variables and can only be applied after the AI system is implemented. Finally, Kaur et al. (2022) [2] have done an extensive survey analysis of the requirements for a trustworthy AI. However, it does not specify how to operationalize the desired principles for a trustworthy AI and does not validate those principles with different stakeholders. We build upon the suggestion of Kaur et al. (2022) [2] of the need for an expectation management framework for AI systems and its evaluation with different stakeholders. This framework is necessary to clarify what various stakeholders expect from the system prior to any data collection, modeling, or implementation, thereby avoiding inflated or low expectations and increasing users’ acceptance rate and trust in the system.

This study introduces an expectation management framework for AI project creation, drawing on the foundational principles of trustworthy AI and theoretical insights into user acceptance and technology use. The framework was validated through the solicitation of end-user feedback via semi-structured interviews in healthcare and education domains. Analyzing the feedback through qualitative systematic text analysis using a deductive category methodology revealed key insights. This framework serves as a valuable tool for policymakers, guiding them in shaping policies related to designing and implementing AI systems in healthcare and education. Additionally, it provides a practical resource for designers of trustworthy AI systems, aiding them in conducting interviews to extract end-user expectations that significantly influence trust and acceptance. The study also contributes by summarizing specific end-user expectations for AI systems in healthcare and education.

This paper is organized as follows: Section 2 discusses this study's Theoretical foundations and concepts. Section 3 describes the methodology used in this study. Section 4 discusses Testing the proposed framework in the Education and Health sectors. Section 5 presents the discussion, and Section 6 the implication of the study. Finally, Section 7 presents the limitations and future work, and Section 8 concludes the paper.

## 2 Theoretical foundations of the AI expectations management framework

### 2.1 Drivers of a trustworthy AI

The use of trustworthy AI is emerging as a strategy to manage expectations and improve system acceptance. Due to the high-stakes uses of AI systems in the public and private sectors, it has become important to make these systems trustworthy, not just to encourage adoption and acceptance through clear expectation management but to ensure the safety of those affected by the systems as well. Trustworthy AI has been gaining attention from organizations such as the International Organization for Standardization [14], the National Institute of Standards and Technology [15], the European Union (EU) [16], and the United States Government Accountability Office [17] among others, as key to ensuring that the issues associated with AI are addressed.

These and other organizations have established ethical guidelines for the development of AI systems [18]. Based on research comparing the various proposals, five main principles have been identified as being present most frequently: transparency and explainability, justice and fairness, non-maleficence including societal and environmental well-being, responsibility and accountability, and privacy [19,20]. Building on the review paper of Kaur et al. (2022) [2], in this study, the EU guidelines for trustworthy AI are used, as they include these five principles as well as other human-centered aspects [16]. The EU guidelines recommend that trustworthy AI should be lawful, ethical, and robust by adhering to the following seven key requirements: human agency and oversight, technical robustness and safety, privacy and data governance, transparency, diversity, non-discrimination and fairness, societal and environmental wellbeing, and accountability. In addition to these seven key requirements, this framework incorporates individuals’ acceptance and adoption of AI systems. Acceptance and adoption of technology, as well as the reason for its inclusion, are described in Section 2.2, while expectations related to trustworthy AI follow presently.

#### 2.1.1 Human agency and oversight

The concept of human agency and oversight in trustworthy AI is based on the potential risk to individuals, society, and the environment when AI is autonomous, rather than when used as a complement to human decision making. It is predicated on the idea that systems can make decisions and take actions that are unexpected due to biased data or errors in the algorithms, and those decisions may have significant consequences to either individuals or to society as a whole. Researchers have shown that fear of loss of control of AI systems is a significant factor in the rejection of AI technology, as individuals do not trust systems that lack human involvement [21]. Human oversight can identify and correct errors, which may allow mitigation of the negative effects of these systems. In addition, a method in which collaboration between humans and AI systems is used has the capacity to outperform methods in which humans alone or AI systems alone are used [22].

Systems with different risk levels require different levels of human involvement [2]. If the system deals with lower levels of risk, human involvement is less important. An example of a system like this would be a product recommendation system – while AI is used to decide which products are recommended to a customer on a company's website, the risk to an individual who receives a poor recommendation is minimal. In contrast, systems dealing with life and death situations require a higher level of human involvement. An example of this type of system is Watson, which can recommend disease treatment plans [23]. Receiving a poor recommendation, in this case, has the potential for great harm to an individual.

Various approaches to human oversight and agency have been proposed. Kaur et al. (2022) [2] define four levels of risk and resultant human involvement requirements, ranging from systems with high levels of human involvement and high risk to fully autonomous systems with less risk. A taxonomy involving both the level of autonomy and the level of automation was developed to categorize human-machine collaboration, with the two axes of the taxonomy ranging from deterministic (unadaptable, transparent) systems to open systems (adaptable, non-transparent) and systems that offer recommendations (high human involvement) to fully automated systems [24]. Other researchers have recommended that enabling end-users to rapidly contest an AI system's decision they believe is faulty is an important way to ensure that proper levels of human oversight and agency have been incorporated [25].

#### 2.1.2 Privacy and data governance

The importance of privacy in AI systems is based on the considerable risk of misuse of the vast amounts of data required by those systems to make decisions. Researchers have shown that expectations of a loss of privacy due to data needs of AI systems are a significant factor in the rejection of AI technology, as individuals do not trust systems that are vulnerable to data breaches or systems from companies that use their data for purposes other than the explicitly stated use [26].

Several examples of AI privacy violations leading to negative consequences for end users are available in the literature. The most salient of these consequences are financial fraud, identity theft, and discrimination, but there are other less anticipated consequences as well. The social networking site Facebook provided the personal information of its 50 million users without their consent to Cambridge Analytica, which then used that data to manipulate the United States presidential elections of 2016 [27]. DeepMind used patient data to assist in the management of acute kidney injury for the Royal Free London NHS Foundation, but patients did not have control over how their data was used, privacy impacts were not adequately disclosed, and patient data was subsequently transferred from the United Kingdom to the United States [28]. The EU has recognized privacy as a human right and has implemented the European General Data Protection Regulation (GDPR) in part to safeguard citizens’ data from misuse [29].

Many methods are available for addressing privacy and ensuring proper data governance. For example, in searchable symmetric encryption, training data can be indexed for keywords or other identifiers and encrypted into unreadable formats, which allows for search on the index and then decryption of matching results for training or training on encrypted data [30].

#### 2.1.3 Transparency, explainability, and interpretability

Interpretability in AI systems refers to the ability to translate the principles and outcomes of the system in a way that is understandable to humans, and that does not affect the system's validity, which is necessary due to the complex nature of some machine learning algorithms [13,31]. A lack of understanding of models can lead to mistrust of the system due to an inability to identify and correct errors or bias and an inability to determine if the system is being used ethically. While transparent models such as decision trees, linear regressions, rule-based models, tree-based ensemble models, and classification rules are readily understood and reproducible, it is not possible to know how the output of the system is reached with so-called black-box models without additional measures being taken [32].

Explainability refers to a branch of AI focused on generating explanations for these complex systems to ensure that the various stakeholders using the system, or those who are impacted by its decisions, clearly understand its performance and limitations. A study by Shin (2021) [9] with 350 subjects relating to news article recommendation services found that explanations of why certain articles are recommended generate trust in the AI system and that user understanding of the explanation provides them with emotional confidence in using the system. This example of explainable artificial intelligence (XAI) is one in a growing area of research in the field of data science. Many methods are available for designers to employ [[33], [34], [35]], for example using visualization-based proxy models on top of black-box models can provide a visual model of the internal workings of the AI system, although they cannot be used with extremely complex models [36].

Considering the fast expansion of AI techniques in all aspects of human life, it is increasingly important to use explainable AI to detect unfair or unethical algorithms. Many examples highlight the importance of having fairer and more transparent AI systems, as we have witnessed the detrimental consequences of algorithms exhibiting gender and racial bias [37].

While there is no universal classification of XAI approaches, they can be divided into two broad categories: (i) methods that directly communicate how the model works, known as transparency, and (ii) methods that explain how and why the model reached certain conclusions, known as a post hoc method [38]. A transparent model can be seen as one that is easy to understand immediately, while post-hoc explainability provides understandable information about how a pre-existing model generates predictions for a given input [39].

There is a heated discussion about the increased complexity of artificial neural network (ANN) models and their decreased interpretability. The extensive use of ANN has led to the emergence of so-called black-box models, including deep learning and ensembles. This does not mean that AI is completely opaque – we should keep in mind that there are so-called white-box or glass-box models that produce easily explainable results – common examples include linear and decision tree-based models. However, ANN models, especially deep learning, are now dominant.

XAI techniques are currently able to provide different types of explanations to facilitate the interpretation of complex systems. One possible categorization identifies six types of explanations that XAI provides: global explanations, local explanations, contrastive explanations, hypothetical explanations, counterfactual explanations, and example-based explanations.

As far as explaining any black box model is concerned, LIME [40] and SHAP [41] are the most comprehensive and dominant methods in the literature for visualizing interactions and feature importance. LIME and SHAP methods have been shown to be applicable to any type of data but not model agnostic. Friedman's Partial Dependence Plots (PDP) [42], although a much older model and not as sophisticated, remains important. It has been very difficult to build high-performance white-box models, especially in natural language processing and computer vision, where the performance gap with deep learning models is unbeatable. Moreover, white-box models, which typically perform well on only one specific task (AI models are expected to be competitive in more than one domain), lose traction in literature and quickly fall behind in terms of interest.

#### 2.1.4 Diversity, non-discrimination, fairness, bias mitigation

Diversity, non-discrimination, fairness, and bias mitigation are a group of related topics relevant to trustworthiness as AI systems can perpetuate and amplify existing biases, whether present in the data used to train the system models, the algorithm's handling of the data, or even the systems in place to monitor the system. These biases can lead to decisions that disproportionately negatively affect marginalized groups in society. Bias mitigation is necessary to identify issues with the data itself or with the algorithm's handling of the data and to adjust the outcomes of the systems accordingly so that the ultimate decisions are fair and representative.

Data bias occurs either when the data used to train the AI model does not provide an accurate representation of the population or when human biases against groups of people are present in the data. One example of data bias in which the data does not provide an accurate representation of the population was found in a scoping literature review by Daneshjou et al. (2021) [43], wherein over 100,000 images used to develop or test clinical AI algorithms in dermatology, only 20% included descriptions of ethnicity or race, and only 10% included information about skin tone. This lack of transparency leads to conditions in which data bias is likely, as there is no way to determine if a diverse patient set was used to develop the algorithms.

Model bias occurs when the algorithm does not capture the true logic of the prediction problem. An analysis of a widely used patient risk score algorithm showed significant racial bias introduced by the model, which scored black patients as high risk only when significantly sicker than white patients [44]. Since the model was basing its prediction on reimbursement/cost, which is a proxy for access to benefits, and since access to healthcare in the United States is unequal, using that dimension as the basis for prediction led to biased results. In relation to model bias, evaluation bias can occur when an incorrect metric is used to determine how well the system performs [45].

Identifying bias in an AI system aims to address it so that the system produces fair results. Addressing fairness in AI systems can be complicated, though, as what is considered fair can vary among individuals and groups [46]. Numerous algorithms to identify and remedy bias in AI systems to achieve fairness have been published; for example, a prejudice remover technique in which predictions are made independently of a protected attribute can be used [47]. Taken as a whole, these techniques are designed to increase fairness in AI algorithms through group/subgroup fairness, in which different demographic groups are treated similarly by the model, and through individual fairness, in which similar individuals are treated similarly by the model.

Mitigating bias in data science, particularly in the design of AI systems, is essential for ensuring fairness and ethical decision-making. Human supervision throughout the AI development process is crucial. Experts, with their deep understanding of societal norms and contexts, can identify and rectify subtle biases that might be missed by automated systems [48]. Moreover, data pre-processing is a pivotal step. Before using data in machine learning algorithms, it must be rigorously examined and cleansed of inherent biases. By integrating meticulous human oversight with thorough data pre-processing, we can edge closer to developing AI systems that are both potent and unbiased.

#### 2.1.5 Accountability

Algorithmic accountability involves assigning responsibility when the AI system causes harm and the need to ensure the actions and decisions of the AI system adhere to legal and ethical standards. Researchers have shown that the lack of accountability of AI systems is a significant factor in the rejection of AI technology, as individuals are more likely to accept systems in which accountability for the system's actions and decisions is assigned [49].

Failures of AI systems have caused severe harm. For example, two fatal crashes involving the Boeing 737-MAX aircraft's automated system led to the death of 346 people [50]. In 2018, Elaine Herzberg became the first pedestrian to die after being hit by a self-driving car [51]. A recent systematic review of accountability in AI systems identified numerous difficulties with assigning accountability, among them the issue that many people work to design the algorithms, the problem that black-box algorithms do not show their decision-making processes, the confusion about what needs to be accounted for, and the lack of ability to enforce consequences [52]. There is an expectation from end users, managing entities, and governmental organizations that AI systems are used responsibly, that errors or biases can be remedied, and that the systems align with legal requirements and ethical guidelines.

Many of the strategies discussed earlier in this literature review, such as using AI systems to assist with decision making rather than being autonomous, setting appropriate evaluation metrics for the problem requirements, utilizing unbiased data and unbiased algorithms, and employing various interpretability and explainability methods not only address the expectations categorized in the sections in which they were mentioned but ensure accountability as well. In addition, a legal framework should be used to ensure the model works within the required boundaries. Although trade secrecy laws may limit the requirement for private companies to disclose their algorithms, whistleblower protection laws have the potential to be used as a means to enforce accountability, as whistleblowers can shed light on potential biases, errors in algorithms, or unethical practices [53].

Impact assessments should be completed to determine who will be directly or indirectly affected by the system and to what extent. Several facets should be explored, including conducting one or more of the following impact assessments: human rights, environmental, privacy, data protection, surveillance, ethical, or algorithmic [54]. A conformity assessment, as proposed by the EU in the Regulation on Artificial Intelligence initiative, may also be required if the AI system poses a high risk to the safety or rights of the individuals they affect [55].

Auditing is an additional way to ensure accountability in AI systems. Ethical algorithmic auditing can be used to measure the ethically salient features of an algorithm and compare that to relevant stakeholder rights and interests, for instance Ref. [56].

#### 2.1.6 Technically robust and safe

Technical robustness and safety refer to the need to ensure an AI system is working as intended while addressing two issues: the expectation that the AI system will function correctly even when faced with unexpected inputs and resilience to malicious attacks. Thorough testing and evaluation before deployment allow designers to determine if there are any issues with how the system is functioning, the accuracy of results it is producing, and if its decisions are harmful in any way before it is used In a live environment. In some cases, there is no mechanism to test whether the system is working correctly, as the results should be unknown. This is known as the oracle problem [57]. The solution typically requires extensive human involvement, although some oracle tests have been developed [57]. For instance, researchers from Carnegie Mellon developed Automated Stress Testing for Autonomy Architectures (ASTAA), in which unexpected values are generated and test cases with unexpected messages, which are more likely given the distributed nature of autonomous systems [58]. The importance of AI systems functioning correctly even when faced with unexpected inputs, as is often found in real-world environments, can be illustrated through the consideration of autonomous vehicles. For example, although expectations that the use of autonomous vehicles can increase safety are high [59], this is also an area where fatal errors can occur because of unexpected real-world environments that differ from training and testing, as in the Elaine Herzberg case described previously [51].

The second issue that falls under the category of robustness and safety is resilience to malicious attacks. AI systems can be uniquely susceptible to malicious attacks due to the large amounts of data the systems use. Hackers can target the data sources in various ways to manipulate the predictions, often over a period of time, so that the effects are not immediately discernible. Some attacks could be as simple as placing small stickers on a stop sign to cause an autonomous vehicle to misinterpret it [60]. Attacks on AI systems can be categorized into input attacks, in which data fed into an existing system is manipulated to change the output of the system to benefit the attacker, and poisoning attacks, which occur when the system is being developed and thereby damage the model itself, creating a backdoor through which the attacker can control the system's output [61].

To address resilience to malicious attacks, Comiter (2019) [61] proposes using an AI Security Compliance program that encourages adopting best practices. Other researchers recommend using an application that ensures all records and operations performed by the AI system are logged and can be audited, that strong access control and authentication are implemented, that sensitive data is sanitized, and that unauthorized access is detected [62].

#### 2.1.7 Non-maleficence, societal and environmental wellbeing

Non-maleficence and societal and environmental well-being have emerged as concerns since AI systems have the potential to amplify inequities in society, disproportionately negatively affecting vulnerable groups. While the previously discussed negative societal impacts, such as loss of privacy, safety concerns, and difficulty assigning accountability, are expected, job displacement is also a growing concern. AI systems can automate many tasks, leading to job losses and increased economic inequality, especially for low and mid-level workers. This could, in turn, lead to more individuals in need of government support services, with fewer working individuals providing revenue for the government in the form of taxes. Several solutions to this issue have been proposed, including workforce retraining to give workers the required digital skills for employment [3], welfare reforms, such as the implementation of a Universal Basic Income to ensure that all individuals whose jobs are impacted by automation are able to afford essentials such as shelter and food [63], and disclosure of the savings made in social security due to automation, where corporations would pay the difference to allow for extended welfare programs [3]. In anticipation of this, designers of AI systems can conduct impact assessments, as discussed earlier in this literature review.

AI-generated art and music raises questions about authorship, copyright protections, and the value of human creativity. The data used as training material for generative AI systems like these are typically copyrighted paintings, photographs, drawings, or musical compositions. Copyright law has not yet addressed the issues of authorship or ownership in AI-generated art or liability and sanctions for copyright infringements [64].

From an environmental perspective, AI systems have the potential to benefit many areas related to well-being, including sustainable food production, access to potable water, and green energy generation and usage [65]. A recent review of progress toward the Sustainable Development Goals showed that these potential benefits are not certain, in that although there are growing expectations, the current landscape is still in the early stages [66]. AI systems have also been shown to actively work against environmental well-being, namely when Volkswagen designed its AI system to fraudulently allow vehicles to pass emissions testing by reducing nitrogen oxide emissions during the test [67].

### 2.2 Acceptance and adoption of AI: beyond trustworthiness

Aside from the expectations of trustworthy AI systems described in section 2.1, there are more general expectations related to the acceptance of AI that has been used to formulate this framework. The Unified Theory of Acceptance and Use of Technology (UTAUT) posits that user intentions to accept and use an information system are influenced by four main constructs: performance expectancy, effort expectancy, social influence, and facilitating conditions [68]. In this model, users’ age, gender, and experiences fall under the construct of enabling conditions and can impact the acceptance of technology.

Practically, many of these constructs are beyond the control of the designer of an AI system. Social influence and enabling conditions are more related to the environment in which the AI system will be used and to the individuals who will be using the system. While a designer should consider these factors before attempting to build a system, only proceeding if conditions are in place in which the system will be successful, the system itself cannot address social influence or enabling conditions. Performance expectancy and effort expectancy measure constructs that can be influenced directly by the AI system and the system designer. Researchers have recommended several methods to determine user expectations related to performance and effort. Using an AI readiness assessment allows a designer to determine how ready the individual or group may be for the system, that is, determining whether or not the organization can use the AI system for digital transformation [69]. Product feature importance frameworks can be used to determine the most important performance expectations of stakeholders. Examples include the Kano model, in which features of the system are prioritized based on the degree to which they will satisfy the expectations of users, and user-centered agile software development, in which an iterative approach to design with continuous stakeholder involvement is utilized [[70], [71], [72]].

### 2.3 Integrating the theoretical foundations into the AI expectation management framework

AI expectation management framework is presented in Table 1. The framework is based on the main constructs of reliable AI and the theoretical foundations of user acceptance and use of technology. For its construction, the seven principles of trustworthy AI from the European Union and the two principles relating to performance and effort expectancy from the UTAUT as the framework constructs were combined. For each construct, means to operationalize the main objectives were compiled.

**Table 1:** Expectation management framework.

Principles	Objective	Means to Operationalize	References
Human Agency and Oversight	Collaboration level with the AI system	The risk level of the system	[2,24]
		Occurrences of human involvement	
	Monitoring	Frequency of monitoring and re-evaluation	[2]
	Contesting the system	Procedure for redress	[25]
		Handling of errors	
Privacy and Data Governance	Data agreements	How and when end-user data can be used	[14]
	Training and user data protections	Required types of data protections	[2,75]
Transparency, Explainability, and Interpretability	Training dataset information	Data labelling	[2]
		Dataset metadata	
	Model types to use	Use of explainable glass box models	[2,32,36,76]
		Explanatory layers over black box models	
	Metrics for explainability	How well end-users should be able to explain the system	[77,78]
Diversity, Non-discrimination, Fairness, and Bias mitigation	Data corpus	Type of material used to train the system	[79]
		Quality standards for sources of training material	
	Protected classes	Representativeness of training data	[80]
		Type of treatment of protected classes if underrepresented	
	Mitigate bias	Treatment of sensitive attributes	[47,81]
	Fairness	User definition of fairness of the system	[45]
		Group or individual fairness in system decisions	
Accountability	Legal frameworks	Legal requirements in end-user environment	[53]
		Whistleblower protections	
	System audits	How accountability for errors is to be determined	[2,56,82]
Technical robustness and Safety	Testing the system	Metrics for the success of the system	[2]
	Unexpected inputs	Anticipated types of unusual inputs	[58,83]
		System testing requirements	
	Malicious attacks	Protections of system	[62,84]
Societal and environmental wellbeing	Impact of system	Handling of copyrighted information	[54,64,85]
		Impact assessments	
Performance expectancy	Features	Feature/product development framework	[[70], [71], [72]]
		Frequency of end-user consultation during development	
Effort expectancy	Stakeholder readiness	Barriers to the adoption of the system	[69]
	Training and tech support	Technical infrastructures	[68]

Table 2 shows an example of the application of the AI expectations management framework to a specific AI project - the data mining project [73,74]. For each of the six sequential phases of the Cross Industry Standard Process for Data Mining (CRISP-DM), the standard for the development of data mining projects, the set of objectives, and activities that must be considered in order to meet the expectations of stakeholders are identified.

**Table 2:** Framework definitions by phase of development.

Phase of Development	Objective	Definition	Source
Business Understanding and Planning	Collaboration level with the AI system	Higher risk systems require higher levels of human oversight. A taxonomy of automation vs autonomy, as seen in human-machine collaboration literature, can be used.	[2,24]
	Legal frameworks	Laws in place to ensure accountability to the civil rights of the public must be followed. Whistleblower protection policies in place.	[53]
	Impact of system	Royalties to artists, green IS, and impact assessments (human rights, environment, privacy, data protection, surveillance, ethics, algorithmic impact) ensure non-maleficence.	[54,64,85]
	Stakeholder readiness	AI readiness assessment is used to determine if the individual or group can use the AI system for digital transformation.	[69]
	Features	Frameworks such as the Kano model or user-centered agile software development can be used to determine the most important performance expectations of stakeholders; stakeholder feedback is solicited throughout development.	[[70], [71], [72]]
Data Understanding	Data agreements	Data agreements to define how and when data is used in the AI system and by the organization implementing the system allow for awareness of privacy issues.	[14]
	Training dataset information	A summary of data used to train the model using data labeling or a data sheet showing how data was collected and the data's main features increases transparency.	[2]
	Data corpus	Metrics appropriate for the data type used to assess the quality of the corpus.	[79]
	Protected classes	If protected classes are not well represented, data sampling and aggregation methods can be used to reduce bias.	[80]
Data Preparation	Training and user data protections	Masking, pseudonymization, encryption, federated learning, re-identification risk, generative models, and machine unlearning protect data.	[2,75]
Modeling	Mitigate bias	Bias can be mitigated through suppression of sensitive attributes, changing class labels, reweighing/resampling, or using adversarial, interpretable, or independent learning.	[47,81]
	Model types to use	Systems needing high explainability options include decision trees, linear models, rule-based models, tree-based ensemble models, and classification rules. The use of proxy models over black-box models, including feature importance, example-based, rule-based, and visualization-based models, increases explainability.	[2,32,36,76]
Evaluation	Metrics for explainability	Metrics for determining if users understand the system are set with methods such as user-centered approaches, trust mechanisms, or a situational awareness framework.	[77,78]
	Fairness	Ensure fair outcomes for all through counterfactual/awareness/unawareness, demographic or conditional parity, equalized odds/opportunity, or treatment equality.	[45]
	System audits	Ethical algorithmic auditing, code audit, scraping audit, sock puppet audit, collaborative audit, or SMACTR can be used to ensure accountability.	[2,56,82]
	Testing the system	Techniques such as metamorphic testing, expert panels, benchmarking, field trials, and simulated environments can be used to ensure safety.	[2]
	Unexpected inputs	Frameworks such as DeepTest, or invariant testing, such as seen with ASTAA, increase safety by introducing unexpected inputs into the AI system prior to deployment.	[58,83]
Deployment	Training and tech support	Technical infrastructures are in place to support the AI system's end users	[68]
	Monitoring	Having systems in place to monitor and re-evaluate ensures that the AI system continues to function as expected and that no bias has been introduced.	[2]
	Contesting the system	End-user redress allows individuals to dispute the decisions made by the AI system, ensuring increased oversight.	[25]
	Malicious attacks	An AI security compliance program can help prepare for attacks. System logging/audits allow for tracking of attacks. Supplemental AI systems can be used to detect attacks.	[62,84]

## 3 Methodology for using the AI expectations management framework

A set of semi-structured interview questions was self-developed as a collaborative effort with all research team members, as questions to evaluate the framework were not readily available in the literature. To demonstrate the framework's utility in this project, end-user feedback in two domains was solicited: healthcare and education. Please see Appendix A and B for the list of questions and specific scenarios discussed. See Table 3 for a description of the participants' backgrounds.

**Table 3:** Interview subject characteristics.

Participant Number	Group	Degree	Specialty	Years in Field
1	Physician	Doctoral	Internal Medicine	28
2	Physician	Doctoral	Family Medicine	25
3	Physician	Doctoral	Family Medicine	32
4	Physician	Doctoral	Psychiatry	5
5	Physician	Doctoral	Family & Lifestyle Medicine	22
6	Student	Bachelor's	Data Science	4
7	Student	Bachelor's	Data Science	2
8	Student	Master's	English Literature	3
9	Student	Master's	Data Science	1
10	Teacher	Bachelor's	Information Systems	1
11	Teacher	Bachelor's	Music Education	2
12	Teacher	Master's	Special Education	24
13	Teacher	Master's	Elementary Education	18
14	Teacher	Master's	Elementary Education	17

### 3.1 Selection of stakeholders

A recent review of literature on AI in healthcare found that AI systems use has the potential to benefit physicians by relieving duties related to patient records and other administrative tasks, streamlining efforts of physicians through patient screening, advising patients on when to seek help, providing guidance to physicians on diagnosis and treatment options, and reducing medical error or unconscious bias [86]. Different stakeholders in healthcare systems have been shown to have diverse expectations of and opinions on challenges with the adoption of AI systems in healthcare [34,87].

In education, AI has been applied to predict applicant success, identify students at risk of failure early in their studies, personalize instruction, and assess student understanding [[88], [89], [90]]. The increased use of conversational natural language processing AI systems such as ChatGPT by students has resulted in increased enthusiasm for AI use in educational settings, as well as increased concerns about issues such as response quality, plagiarism, cheating, and user privacy [91,92]. Because of the many issues and potential benefits in these fields, end users of AI systems for healthcare and education were selected to validate the framework, as they are likely to have diverse expectations and concerns for the development of AI systems. The feedback from end users was analyzed and used to make adjustments to the framework.

### 3.2 Interview subjects

The protocols for this study met the requirements of the [name deleted to maintain the integrity of the review process] Internal Review Board according to the regulations of the Ethics Committee of [name deleted to maintain the integrity of the review process] with ethical approval reference number of DSCI2023-4-39251. All subjects gave informed consent for inclusion before participating in the study.

Fourteen potential end users were interviewed via phone or in person, depending on the participant's preference and location, to determine whether the framework captured their expectations for a trustworthy AI system. All the interview subjects had at least some experience using an AI system, although not all had used an AI system in their work or studies. Of those who were interviewed, five were physicians, four were students aged 18 or older, and five were teachers. See Table 3 for subject profiles. To elicit opinions on AI systems most likely to be used by these groups, physicians were asked to evaluate a hypothetical decision support system for diagnosis and treatment similar to Merative (formerly IBM Watson Health), whereas educators and students were asked to evaluate a hypothetical system to respond to student questions and requests with detailed responses, similar to ChatGPT. The interview questions are included in Appendices A (Physicians) and B (Teachers and students).

### 3.3 Analysis of interviews

To allow for more accuracy in summarizing the expectations than if notes alone were used, the interviews were recorded and transcribed using Call Recorder, an open-source audio application for android. These transcripts were uploaded to QCAmap, an open-source web-based application for analysis. A qualitative systematic text analysis of interview transcriptions following a deductive category assignment method was used [93]. The deductive category application was chosen as the expectation management framework, and its components are based on straightforward concepts from the previously conducted literature review.

The analytical unit selected was a phrase or clause, which was determined to be the smallest component of the interview material that could be coded sensibly. The recording unit was one document for each interview. Coding decisions were based on the framework constructs and principles based on the literature review, with multiple categorizations allowed if necessary. Each transcribed phrase was read and categorized according to the corresponding means to operationalize the objectives from the framework, with each means being assigned a unique number. Phrases not covered by the framework were coded as “other” and subsequently used to update the framework to be more inclusive of participant feedback. Then, we normalize the phrases per principle to be comparable between the three groups of interviewees; this is, the weight of each principle is calculated as the number of phrases per principle divided by the total number of phrases per group of interviewees (please see Table 4). Common expectations relating to each objective were grouped to identify themes, as reported in Section 4.

**Table 4:** Number of phrases identified by each principle and group of participants (physicians, teachers, and students). Weights are calculated as the number of phrases per principle divided by the total number of phrases.

Principle	Physician Phrases (n = 203)	Teacher Phrases (n = 151)	Student Phrases (n = 120)
Human Agency and Oversight	0.187	0.152	0.117
Accountability	0.069	0.040	0.067
Societal and Environmental Wellbeing	0.099	0.079	0.133
Effort Expectancy	0.010	0.007	0.008
Performance Expectancy	0.227	0.219	0.175
Privacy and Data Governance	0.030	0.079	0.058
Transparency, Explainability, and Interpretability	0.108	0.113	0.192
Diversity, Non-Discrimination, Fairness, and Bias Mitigation	0.103	0.066	0.092
Technical Robustness and Safety	0.054	0.093	0.092
Distrust, Hesitancy	0.113	0.152	0.067

## 4 Testing the AI expectation management framework in the Education and Health sectors

This chapter presents a summary of the feedback from the interview subjects. It is divided into four sections. The first section corresponds to the most common themes heard from the end users in all three subject groups (please see Table 5). The second, third, and fourth sections address feedback and expectations received from the end users, which were unique to each group: physicians (see Table 6), students (see Table 7), and teachers (see Table 8).

**Table 5:** Common expectations among all participant groups.

Group	Results
All Respondents	Ability to contest the system if needed. Data agreements are in place and honored. Ability to know the specific source the system is using. Diverse, quality sources are used to train the system. Regular monitoring of the system by humans, especially in the beginning Protection of personal data from hackers The system is used as a tool, not as a replacement for human reasoning. Concerns that jobs may be replaced by AI systems. Perfect or near-perfect accuracy User input can be used to update the system. Interest in trying or continuing to use the AI system

**Table 6:** Expectations unique to physicians.

Group	Results
Physicians	Specific information about the scientific rigor of sources required - conflicts of interest/funding sources, trial type and size, trial duration, and trial outcomes. HIPAA legal framework used. Physician is ultimately accountable for errors in recommendations. No mention of concerns about language availability Conflicts of interest between the financial interests of the AI system owner and the recommendations the system provides are likely. Negative impact on doctor-patient relationship Physician burnout from additional requirements Not expected to be helpful

**Table 7:** Expectations unique to students.

Group	Results
Students	System will have a positive impact and save time. No mention of concerns about training or technical support

**Table 8:** Expectations unique to teachers.

Group	Results
Teachers	Simple user interface and simple explanations of the system Representation in the system responses should reflect the diversity present in the student population. All students should have equal access. Availability of the system may impact credibility of student work, plagiarism. Alerts for potentially harmful searches

### 4.1 Common expectations among all participant groups

#### 4.1.1 Transparency and explainability

The first general theme to emerge across all end-user groups was the desire to understand the sources from which the AI system derives its information. The following response from Participant 13, when asked if it is important to know where the AI system derives its responses and how much, if any, information they would like about those sources, illustrates the consensus among the end users.

Any time I ask my students to research something, I want to see where they’re getting their research from. So, you know, even if they’ll say Wikipedia, well where did Wikipedia get it? Where’s that breakdown? So yeah, I wouldn’t feel that confident in using [the system] without that … I would want to know where those references are from.

#### 4.1.2 Diversity, non-discrimination, fairness, and bias mitigation

Another expectation that was repeated multiple times across all groups was a desire for sources that used to be credible, free from bias, diverse, and of high quality. The following response from Participant 14 illustrates this expectation.

I would want to know that they are research-based, trusted, yeah trusted-curriculum sort of sources for appropriate age levels. That they are not using random articles online. That they're researched, designed for children. That the information is developmentally appropriate, on grade level.

#### 4.1.3 Performance expectancy

Related to the quality of the sources, users across all groups had an expectation that the AI system would provide information that was near perfect or perfect and, in all cases, better than a human. An example response exemplifying this expectation is from Participant 4.

I would expect it to be as close to 100% accurate as possible. Right? This is the best thing ever. We don’t even need people to think about things, we can just use it. Of course, I’m being sarcastic. But to assume that this is the best of not one journal, but every journal ever written on the subject of ‘blank’ in medicine, and it’s pulling from it, then you would hope it to be quite accurate. As an end user, yes, of course. I mean, it would have to be, otherwise, what are we doing?

#### 4.1.4 Privacy and data governance

Multiple subjects in each group expressed expectations about how data is managed within the AI system. Consensus among the interview subjects is the expectation that data agreements must be in place. The following quote from Participant 7 demonstrates the expectations expressed when asked about privacy.

I would expect it to use the data for what it says it will use the data for.

#### 4.1.5 Technical robustness and safety

In this same vein, although not asked about malicious attacks, several subjects brought up expectations about security. The first excerpt below is from Participant 4 and demonstrates expectations about the vulnerability of personal data to a malicious attack.

If there’s a chance it’s not [secure], or that’s going to be compromised because it’s all going to be connected into one giant source or hive brain, then that should be something that should be proceeded with or stopped. If it’s going to lead to peoples' information being out there. And then also the fear, it’s almost a Hollywood fear, of who then can possibly access that information. And in the United States with health insurance being so closely tied to employment. So. Security is paramount.

The second excerpt seen below is from Participant 9 and demonstrates concerns about the vulnerability of the AI system itself to malicious attacks.

If someone is purposely trying to make the model racist, or discriminative, or doing, I don’t want to say wrong stuff, but socially bad stuff, the model should be protected in regard to that.

#### 4.1.6 Human agency and oversight

Although most subjects expected some degree of human oversight, some groups had stricter expectations and greater concerns about this than others. The universal expectation, however, was that AI systems would be monitored and adjusted as needed, especially at the beginning of their implementation. The following excerpt is from the interview with Participant 3.

There has to be an audit, so to speak, on a, I would say, a regular interval. I could see where it would be hugely helpful in both directions. Like yeah, they were right, or no, this wasn’t right, and this is why it wasn’t right.

When asked what they would expect to happen if they received a recommendation or a response they disagreed with, most interview subjects expected to be able to review the sources the system used to reach its conclusion, ignoring the response if they chose or rephrasing their query if necessary. They also expected to be able to flag the incident so that the system could be updated or could learn from its mistake. This statement from Participant 14 exemplifies the kind of responses received on this subject.

I would expect to be able to somehow inquire as to why that answer is being delivered and also have an avenue to edit it, or petition to have it edited.

#### 4.1.7 Societal and environmental wellbeing

Throughout the interviews, subjects expressed expectations about their preferences and fears of how AI would be utilized. Subjects in all groups expressed that, ideally, AI systems would be available as a tool for humans to use but not as a replacement for humans themselves. This statement by Participant 8 exemplifies the consensus among subjects.

I think that if all these parameters are met and, ethicists have been brought in, and the testing has been done, I could see it being a very effective tool for educating and making life-long learners. A tool. Not the whole thing.

When asked about the potential impact of the hypothetical AI system on themselves or others, several subjects across all groups expressed expectations about jobs being replaced by AI. The following is an example of a statement about this expectation from Participant 14.

They’re saying that that’s where it’s going. Teachers are going to be one of the main professions replaced by AI in the next 20 years. And you can see it because there’s no respect for what we do anymore. It’s on the horizon.

Common to all interview subjects except one was an interest in trying the hypothetical AI system or continuing to use the one they currently have. This was true even in the physician group, which was the least confident that the AI system would improve their practices or be used by physicians if given the option. This statement is from Participant 3.

I think if I had adequate training, and I was confident that it provided all the expectations that you would hope for, that yes, I would use it. If it ultimately leads to better outcomes involved, whether that’s better patient outcomes, or you know, the quadruple aim. Yeah, we’ve got to do something. Our system’s a mess.

#### 4.1.8 Hesitancy and mistrust in AI use

Although outside the scope of the expectation management framework itself, a general distrust of the use of AI systems was expressed by multiple interviewees in each participant group. The following comments are from Physician 2, Teacher 10, and Student 11, respectively:

I believe they studied people and seen that as there’s been more technology, people’s brains have atrophied in some capacity. And similarly, with ChatGPT and other similar types of things we’re seeing just changes with where we’re at. Are we stunting creativity? Are we stunting our ability to just be? Are we using it to make our lives better?

I think it needs to be approached with extreme caution as well. Especially with young learners. You’re shaping young minds. You’re teaching them what it means- and we see this with COVID, right? So essentially society was fractured by COVID. And what we’re seeing now are the effects of that on students and their social-emotional deficits from not being with other kids and not learning, what’s generally the age that they learn, how to problem solve social problems. So, you’re seeing extreme behaviors and extreme issues. So, if you’re taking out this human component to teaching, which part of that is teaching what it is to be human, and you’re putting this artificial intelligence system in place, I don’t know what we 're going to have at the end.

I know that AI is being used in job searching and job applications where an AI program will scan for certain key phrases or words on a resume or on an application or a cover letter, and if it doesn’t find those, you just kind of get dismissed and you don’t then make it to the next level. So potentially I have interacted with it in that way. And if I have, I’m not a fan. Because there’s so much more to an employee or a human than, you know, just keywords. So, yeah. I guess it’s all about efficiency and productivity. Whatever.

### 4.2 Expectations unique to physicians

#### 4.2.1 Accountability

Every physician interviewed expected the AI system to comply with the Health Insurance Portability and Accountability Act of 1996 (HIPAA), a federal law in the United States that regulates, among other things, the use and disclosure of protected health information [94]. Although some members of other groups mentioned legal frameworks, like the GDPR [95], or the restriction on black-box models in certain fields, and many expressed concerns that AI systems are not well regulated, only the physician group universally mentioned a legal framework, and a specific, uniform legal framework. Participant 3 succinctly expressed this expectation.

It has to be HIPAA compliant.

Respondents generally expected that accountability for AI system errors would lie either with the company that created the system or be a shared responsibility between the company and the end user, typically to be determined through an audit. Physicians were unique in that they expected that accountability for errors would, and should, rest with them. Participant 4 succinctly summarized this.

Ultimately, it's the physician in the room with the patient who holds all the responsibility.

#### 4.2.2 Diversity, non-discrimination, fairness, and bias mitigation

Several physicians expressed expectations that the data sources and AI system recommendations may not be trustworthy due to the for-profit healthcare system in the United States. Related to this, some respondents expected specific information about study rigor and quality used to train the AI system to be provided, including conflicts of interest, funding sources, trial type and size, trial duration, and outcomes, including absolute risk reduction. The following is an excerpt from the interview with Participant 5.

Who’s in control of the AI? Is it a private corporation? Because it’s always going to be to grow your business, if that’s it. Or is it going to be a non-profit, and I don’t know that it has to be governmental, but some sort of non-profit motivated organization that is truly controlling AI and that can truly be a free, no ties to any of the pharmaceuticals or other conflicts of interest.

#### 4.2.3 Societal and environmental wellbeing

Additionally, the physicians expressed expectations of a potential negative impact on the doctor-patient relationship. Participant 4 mentions how the introduction of the Electronic Health/Medical Record (EHR/EMR), prevalent at most healthcare facilities in the United States, negatively impacted the ability of doctors to connect with their patients, with similar expectations expressed about a potential AI system.

This is like what we’ve lived through when we see the results of people surveyed when EMR came out. They say, my doctor doesn’t even look at me, he’s just tapping on the computer all the time. That’s what this feels like, again.

Although subjects in the physician group had mixed expectations regarding whether or not an AI system would ultimately be beneficial, expectations about the AI system contributing to physician burnout were prevalent. Related to this was the expectation that physicians may do whatever the AI system recommends, without double-checking sources or accuracy, due to fatigue. The following statement is from Participant 1.

Any time you add that extra piece of technology in, if it’s going to be something that’s actually, something that’s required to use, that you have to click on to get past, or to move into, if you must do what the system is telling you within healthcare, I think that’s rough. And so I just see that as we’re moving forward, burnout for physicians, healthcare providers, increasing, and part of it has to do with all this nonsense.

Although they expected healthcare providers to use the hypothetical AI tool if required, many physicians did not expect it to be helpful at the point of care. Aside from increased workload as described above, physicians cited the diverse factors that impact a treatment plan that is not able to be captured in an electronic system as a reason it may not be helpful. This excerpt is from the interview with Participant 3.

How do you include all the important demographic information and the social determinants of health? And you know there’s just so much to know to really understand the context.

Another reason provided was the expectation that the AI system would not provide novel diagnoses or treatment plans that were not apparent already. This excerpt is from the interview with Participant 4.

If you graduate all the way out and go through, you know you’re an actual MD or a DO, and you go through and you finish your residency, there’s not much you haven’t seen many, many times before. And you know how to do it. This is what conferences, journals, talking to people do if you’re an expert in the field at that point. And that person, say a pediatric oncologist, they have more than the requisite seven years of studying these things. They’ve gone on for probably another five. You know, they’re up to date on this.

#### 4.2.4 Performance expectancy

While an expectation that the AI system should work in multiple languages was expressed by the majority of subjects in both the student and teacher groups, no physicians mentioned this expectation.

### 4.3 Expectations unique to students

#### 4.3.1 Societal and environmental wellbeing

Like subjects in the other groups, some expectations about potential negative impacts were expressed by students. However, individuals in the student group expressed the most positive expectations overall. Positive expected impacts of the system included saving time, compiling study materials from various sources, acting as an assistant, grading papers for teachers, and providing corrective feedback on writing. Participant 9's statement below exemplifies the positive expectations.

In my case, for instance, I am programming. I can save a lot of time searching Stack Overflow if I’m using this tool. And even if the answer is not correct, nobody will die. I am not like discriminating someone, it just goes. And for that, for my specific purpose I could imagine using this or using this like a personal assistant, like, look, just book me this appointment for this date. It could be useful for that. Or send me a recurrent email to this person about this issue. Or give me my shopping list. Things like that could be automated and it’s safe to use for those purposes.

#### 4.3.2 Effort expectancy

No student mentioned an expectation that there should be training or technical support for the hypothetical AI system. This expectation was expressed by the majority of physicians and teachers, even though the structured interview questions did not specifically inquire about expectations for training or technical support.

### 4.4 Expectations unique to teachers

#### 4.4.1 Societal and environmental wellbeing

Teachers expected that a hypothetical AI system used in schools would detect and flag sensitive student inquiries, prompting the teacher to follow up, potentially with mental health professionals. They universally expected that an alert system should be in place for responses the students could use to harm themselves or others. All teachers interviewed expected data agreements to be in place so that students would know how their questions were being monitored. This is an excerpt from the interview with Participant 10.

For the users making the query, this is where it gets difficult. Because it’s like with social media and policing. Where do you draw the line at policing people’s content? Because on the one hand if it’s just neutral queries, that’s all well and good. But if they’re looking for ways, for example, to cause mass destruction, clearly that should be policed. And somehow that should cause a trigger, a flag to be raised for further investigation. But yeah. In terms of privacy, I think there is an expectation of privacy, but with these caveats that there are checks put in place so that potentially harmful behavior can be signaled somehow.

Although teachers expected the hypothetical AI system to help students access information quickly, they also expected students to use the system to write papers for them. Among the three interview groups, this concern was unique to teachers. The following statement is from Participant 11.

I would not know if a paper was written by my student or by AI; and then what does that reflect also on me as a teacher and the community of the students? What do they know? What do they not know? I wouldn’t have that answer.

#### 4.4.2 Transparency, explainability and interpretability

In terms of the algorithms themselves, the interface, and any training, several teachers indicated they would need to be straightforward due to the range of technology literacy in their peers. This statement is from Participant 13.

I would say it would need to be very simple, because most of us using it are not trained in this field. So it needs to be very clear.

#### 4.4.3 Diversity, non-discrimination, fairness, and bias mitigation

Although diversity and lack of bias in sources were expected by most subjects interviewed, unique to the teacher group was the number of comments about expectations of equal access to the system regardless of disability or socioeconomic status, as well as representation of diversity in the AI system responses. This statement from Participant 14 is an example of the expectations expressed.

It would be very important that if there’s visuals used to represent different scenarios that different kinds of people are represented. Different cultures are represented. Different kinds of clothing are represented. Able-bodied and people living with disabilities are represented. That the important thing is that students feel viewed in the curriculum. Right? So, it needs to be as diverse as your student population could ever be … that any pictures of family are not just white people with a boy and a girl and a dog in front of a house. That there’s people that have darker skin and have two dads or have two moms or have a single parent or have grandparents as their parents, and they live in different houses, and those kinds of things.

## 5 Discussion

The framework is intended for use as a tool to capture and understand the expectations of stakeholders at different phases of AI system development. Interview subjects were selected from diverse disciplines to demonstrate the utility of the framework. Prior to collaboration between disciplines or industries, the framework can be used to ensure a clear understanding of what the stakeholders expect.

Interviews were conducted to solicit expectations of trustworthy AI systems. Common expectations of the three end-user groups included knowledge of the sources of the AI system, diverse and quality sources being used, perfect or near-perfect accuracy of the system, the use of data agreements and the protection of personal data from malicious attacks, regular monitoring of the AI system, an ability to contest incorrect responses from the system, using input about incorrect responses to update the system, and various concerns about the impact of the system, including a concern that jobs will be replaced by AI systems. Several users had unique ideas in terms of the functionality of the system or features they would like to see implemented. All interview subjects except one were interested in trying or continuing to use the AI system.

The use of the expectation management framework led to the capture of a wide range of potential end-user expectations. In the cases of the three end-user groups interviewed, several common expectations were captured, but the framework was also able to capture unique expectations specific to each group. In addition to this, individual expectations not common to other members of the group were captured.

Results are consistent with the trustworthy AI constructs present in the literature, namely in the recent review article on trustworthy AI [2] and the EU's trustworthy AI constructs [55]. End users had expectations related to a hypothetical AI system from each of these constructs and from the constructs in the framework that are applicable to AI designers from the UTAUT [68].

The results support asking end users, in simplified terms that individuals not in the field of data science can understand, about their expectations related to each of the principles in the framework. While many of the principles in the framework contain techniques that an individual from a field such as education or healthcare would not be familiar with, the expectations surrounding those principles are addressed in using them. For example, while reweighing or resampling data may not be a technique that end users are familiar with, they do have an expectation that any bias in the source data is mitigated. The specific techniques used will change based on the current state of the art, but the expectation of unbiased models will likely be consistent over time.

Based on the literature, the following trends in this field can be identified: the use of AI is increasing [[96], [97], [98]], the expectations of AI are increasing [21,99,100], and the governance of AI is lagging [53,54,64,101]. From these trends, we can infer that designers of AI systems have an acute and vital need to capture user expectations in detail prior to embarking on any project. An expectation management framework such as this one must be implemented to ensure the creation of AI systems that are trustworthy and safe.

A current gap in the literature includes an expectation management framework to be used during the design, development, and adoption of an AI system [2]. This can help ensure trustworthiness and explainability so that stakeholders understand the shortcomings of the models, what information can be trusted and to what extent, generally how the algorithm works, what the data sources are, how the system handles important issues such as fairness, bias, and privacy, what regulations are in place for the model, and how it is being monitored and maintained. Other gaps include how best to address data literacy and tech literacy related to AI in the general population [102,103].

### 5.1 Potential issues with point-of-care healthcare applications

In general, subjects in the physician group had the lowest expectations of the system being helpful. Many expected that the system would negatively impact the doctor-patient relationship and become an extra burden on physicians that would contribute to physician burnout. They had concerns about conflicts of interest in recommendations and thus required specific information about the scientific rigor of the sources used to train the system. They had an expectation that the system be HIPAA compliant. Unlike the student and teacher groups, the physician group did not expect the system to be available in multiple languages. Also, unlike the student and teacher groups, the physician group expected that any errors in recommendations from the system were ultimately the responsibility of the physician.

An important issue brought to light through the use of the framework in this study was the general hesitancy of the physician group. Many physicians expected that the AI system would be unable to provide diagnosis or treatment options that they were not already aware of. In addition, they expressed expectations that conflicts of interest in recommendations were likely. Pairing these expectations with their expectation that, like the previously introduced technology of the EHR, the system would be purported to save time but would, in fact, do the opposite, one could logically infer that a point-of-care AI system would not be adopted. Physician frustration, lack of integration into the EHR, and incomplete training data, all concerns brought up by participants in this study, were also contributing factors to the failure of IBM Watson Health, a more than $5 billion investment that was recently sold for approximately $1 billion [104]. Using a framework such as this has the potential to obviate unnecessary large investments in systems that will be poorly received. Instead of point-of-care systems, artificial intelligence has greater potential to help the field of medicine with analyzing large datasets in research settings [[105], [106], [107]].

### 5.2 Inclusivity, access, and explainability in education applications

Students, in general, had expectations that were most consistent with other groups. They were less concerned about training and technical support than both physicians and teachers. The student group was the most likely to expect the AI system to have a positive impact, citing several advantages, especially the ability to save time when using the system. Subjects in the teacher group had expectations specific to their needs, including alerts about student questions surrounding potentially dangerous topics such as weapons or suicide. They had an expectation that answers and content should reflect the diversity of their student population and that all students should have equal access to the system, regardless of socioeconomic status or disability status. They expected simple explanations of the system. They also had unique concerns about the issues of plagiarism and the credibility of student work.

It is important to note that the expectations of many users in this study are incompatible with the AI technologies they are using. For example, all users expected to see the exact sources the hypothetical AI system used to arrive at its answer. This expectation was especially emphasized by the education end users. While providing an exact singular data source might be possible for some models, it is incompatible with generative language models such as ChatGPT. Using an expectation management framework such as this one is essential in uncovering these issues before designing the system.

## 6 Implications

This study's participants expressed positive and negative expectations as described above. However, the framework itself is a neutral tool to determine existing expectations. How the framework results are applied will result in either positive or negative outcomes. Risks in misapplying or ignoring expressed concerns from participants include autonomous systems making incorrect or discriminatorily biased decisions, lack of accountability for errors, negative impacts on human rights and the environment, systems that are unused due to lack of valued features, misunderstanding of systems and their limits by end-users, and algorithms that work against the interest of the end users. Conversely, careful application of the means to operationalize the framework principles can help mitigate these potential negative impacts.

### 6.1 Implications for society

Lawmakers can take advantage of this framework to guide policymaking regarding the design and use of AI systems in the healthcare sector to minimize potential negative impacts and increase the trustworthiness of these systems. Specific issues revealed that are important for policymakers to address include potential bias of source data, security of protected health information, and conflicts of interest in both studies used as source data and in treatment recommendations provided by the system.

Lawmakers can also use this framework to guide policymaking for AI systems in education, ensuring end-user understanding of both benefits and limitations. Specific issues include requirements for explainability layers regarding how the system functions and how it arrives at its responses and the need to ensure equal access to systems irrespective of disability, socioeconomic status, or other disadvantaged group characteristics.

Common expectations across healthcare and education end users suggest similar issues will likely arise across various sectors of society. Policies addressing issues created by the use of AI systems are needed [101]. Based on the framework and its validation, these issues include the impact on the workforce, privacy, misinformation, or information derived from systems with conflicts of interest, safety, discrimination, human oversight, explainability and data literacy, and accountability, among others.

### 6.2 Managerial implications

The framework can be used as a roadmap for designers of trustworthy AI systems in the health and education sectors when conducting interviews with end users to ensure a rich discussion of their expectations. Doing so can provide insights into the importance of various features of the system, provide a picture of the end-user understanding of the technology, and provide insight into the kind of explanatory layers and training that may be necessary once the system is in production. Using the framework is a simple way to identify potential issues that will eliminate unnecessary expenses in creating the system itself.

In the healthcare sector, development related to trustworthy AI systems can focus on processing large amounts of data in research settings rather than point-of-care systems. Ensuring unbiased data is used to train the potential systems is of utmost importance, given the high stakes of the applications. In the education sector, development related to trustworthy AI systems can be focused on enhancing the availability of systems in languages other than English and ensuring proper explainability of the systems.

### 6.3 How to use the AI expectation management framework in each phase of development

**Fig. 1:**
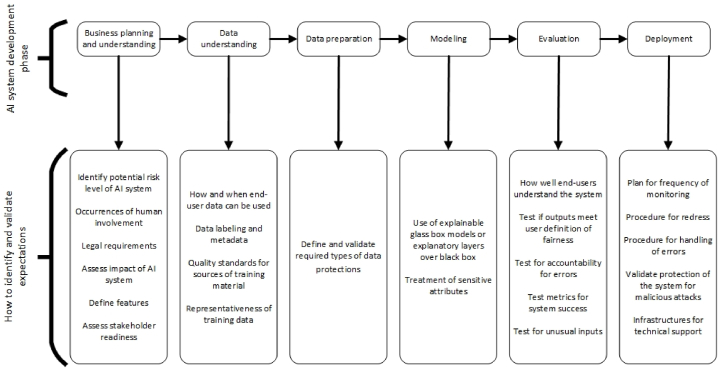
Workflow of the AI expectation management framework use.

The main goal of the AI expectation management framework is to guide the design, development, and deployment of AI systems that can achieve the trustworthiness of its stakeholders and end-users. The authors propose to use the framework following four main steps: i) Use the principles of the framework as a guide to understanding what stakeholders expect in terms of human agency and oversight, privacy and data governance, transparency, explainability and interpretability, diversity and fairness, accountability, technical robustness and safety, societal and environmental wellbeing, performance expectancy, and effort expectancy. ii) For each principle, determine the means to operationalize it. This involves translating the principle into concrete actions or procedures that can be implemented during the development and deployment of the AI system. For example, under the principle of ‘Privacy and Data governance,’ operationalizing could involve establishing data agreements and determining the required types of data protection. iii) The framework should be used iteratively throughout the AI system's lifecycle. After the initial identification of expectations and operationalization of principles, gather feedback from stakeholders and adjust the system accordingly. This iterative process helps to ensure that the system continues to meet stakeholder expectations as it evolves. Finally, iv) Document how the principles have been operationalized and any changes made based on feedback. This documentation is important for transparency and accountability. It's also crucial to communicate with stakeholders throughout the process, keeping them informed about how their expectations are being met and any changes that are made. Fig. 1 presents a workflow of the development phases and the means to identify and validate the expectations.

## 7 Limitations and future work

There are several limitations to this study that can be addressed in future research. The first is that this is a qualitative study with a fairly small sample size of fourteen interviews. The types of end users were limited to only physicians, students, and teachers, so results may not be generalizable to other industries. Future research should aim for a more diverse sample to enhance the external validity of the results. In addition, the differing levels of familiarity with AI in the interview participants likely impacted their responses.

Second, besides healthcare and education industries, future studies could validate the framework on additional real-world applications to strengthen its credibility and further explore expectations of end users, such as construction, marketing, or retail. To conduct the validation, future studies may adapt the questionnaires available in Appendix A and B to conduct the interviews in different settings. The need to expand the framework to use with other stakeholders, apart from end users, also exists. A future study might consider whether changes to the framework would be needed when used with stakeholders such as administrators and policymakers.

Third, although the study explains how to use the proposed framework throughout the AI system's lifecycle in section 6.3, it is not possible to fully predict how effective the results from the framework will be on real-world applications. Future research may further validate the proposed expectations management framework covering the whole AI system's lifecycle, at least in a pilot study or lab environment.

Finally, this study was completed at a time when the AI system ChatGPT was increasing rapidly in terms of user interest and media coverage. While this may have helped the interview subjects be more familiar with the potential applications of AI, it also may have led to many participants feeling that it was important to express their hesitancy and distrust of AI in general, raising concerns about the temporal sensitivity of the findings. This limitation may be addressed in future studies using a longitudinal data collection to test for potential temporal sensitivity of the findings.

## 8 Conclusions

In our study, we developed and validated an expectation management framework for trustworthy AI systems through semi-structured interviews with potential end-users in the healthcare and education sectors. This framework serves as a tool to capture users' expectations effectively. The application of this framework exposed a wide range of end-user expectations, revealing both common and unique anticipations among different groups and individuals. Shared expectations included transparent source disclosure, data protection, accuracy, regular monitoring, and an avenue for contesting system errors. Interestingly, educational users seemed to expect more benefits from a generative language AI system than healthcare users did from a point-of-care AI system. Healthcare users uniquely expected HIPAA compliance and had concerns about data training rigor, potential physician burnout, and potential impact on the doctor-patient relationship. Meanwhile, educational users expressed desires for multi-language availability, harmful search alerts, and representation but had concerns about plagiarism and credibility. These insights underscore the importance of using the framework to capture all trust-related expectations before designing and implementing any AI system. This dialogue assists in understanding key user expectations, the importance of system features, and potential issues beforehand. Furthermore, it offers insight into the necessary explanatory layers and training once the system is in production, thereby supporting the construction of a truly trustworthy AI system.

## Data availability statement

The data that has been used is confidential.

## Ethics declarations

This study was reviewed and approved by the NOVA IMS ethics committee, with the approval number: DSCI2023-4-39251.

All participants provided informed consent to participate in the study.

## Consent to publish

All of the authors consented to publish this manuscript.

## Funding

This work was supported by funds of Fundação para a Ciência e a Tecnologia (10.13039/501100001871FCT) through a research grant to the Information Management Research Center – MagIC/10.13039/501100005855NOVA IMS (UIDB/04152/2020).

## CRediT authorship contribution statement

**Marjorie Kinney:** Writing – review & editing, Writing – original draft, Validation, Methodology, Investigation, Formal analysis, Data curation, Conceptualization. **Maria Anastasiadou:** Writing – review & editing, Validation, Supervision, Methodology, Conceptualization. **Mijail Naranjo-Zolotov:** Writing – review & editing, Validation, Supervision, Methodology, Conceptualization. **Vitor Santos:** Writing – review & editing, Validation.

## Declaration of competing interest

The authors declare that they have no known competing financial interests or personal relationships that could have appeared to influence the work reported in this paper.

## APPENDIX A

### Semi-Structured Interview Questions – Healthcare End Users

#### Demographics

1) Name
2) Education level
3) Profession
4) Medical specialty (if applicable)
5) Years of working in this field

#### Familiarity with AI

1) How would you define artificial intelligence?
2) Is there an AI system for medical diagnosis or treatment at your facility?
3) Have you used an AI system in your work before? If so, please describe the system (what is it used for, who is using it, and how useful you think it is).

### Introduction to AI (can omit if subject understands AI)

“Although there are differing definitions of artificial intelligence, in general, and for the purposes of this study, we will define artificial intelligence (AI) as the simulation of human thinking, reasoning, learning, and problem-solving. It can also refer to a machine or a computer program that mimics humans or their actions.”

### Introduction to AI System (adapted from IBM website, can omit if subject understands AI in healthcare systems)

“Please imagine that a clinical decision support system that leverages AI to provide information that aids the efficient delivery of personalized, evidence-based care is being designed for use at your facility. An example of a system like this is IBM Watson Health (now known as Merative, which includes products like Micromedex). Consider what your expectations are related to the system that is being developed.”

#### Questions about the framework (may omit any if already addressed in previous responses)

1) What would you expect the main goals or outcomes of this system to be?
2) Human oversight refers to the idea that the responses and functioning of the AI system are monitored by humans rather than having a system that functions completely independently. How much, if any, human monitoring would you expect for this system?
3) What would make this system easy to use?
4) What sort of information would you expect to be given about the patient data that is used to create this system?
5) How diverse would you expect the patient data used to create this system to be?
6) What are your expectations regarding patient privacy in this system?
7) Different AI systems have different levels of accuracy. This can depend on many factors and can change within the same AI system over time. How would you know if this system is performing well?
8) How well do you expect this system to work?
9) How often would you expect the system to be evaluated for accuracy?
10) What would make this system fair to use with all patients?
11) Do you expect the AI system to self-explain its algorithms? If so, what kind of explanations would you need in order to understand the logic of the algorithm?
12) What would you expect to happen if the system receives unexpected patient information?
13) What would you expect to happen if you received a suggestion you disagreed with?
14) Who should be accountable for errors in recommendations from the system?
15) What sort of impact do you expect this system to have on your patients and/or your facility?
16) Are there any other expectations you would have for this system that haven't been covered by these questions?
17) Do you anticipate that you would use this kind of system? Why or why not?

#### Discussion of the principles

“I am going to show you a list of principles the designer of an AI system might use when building it. You may have mentioned some of these principles in our discussion already, and others might be things we have not yet talked about. Are there any principles you would expect the designer to use that you have not mentioned already and that are not included in this list?”

## APPENDIX B

### Semi-Structured Interview Questions –Education End Users

#### Demographics

1) Name
2) Education level
3) Profession (if applicable)
4) Educational discipline (if applicable)
5) Years of working in this field (if applicable)

#### Familiarity with AI

1) How would you define artificial intelligence?
2) Have you used an AI system in your work or studies before? If so, please describe the system (what it used for it is used for, who is using it, and how useful you think it is).

### Introduction to AI (can omit if subject understands AI)

“Although there are differing definitions of artificial intelligence, in general, and for the purposes of this study, we will define artificial intelligence (AI) as the simulation of human thinking, reasoning, learning, and problem-solving. It can also refer to a machine or a computer program that mimics humans or their actions.”

### Introduction to AI System (adapted from OpenAI/ChatGPT website)

“Please imagine that a system is being developed that will use AI to respond to student questions with detailed responses. An example of a system like this is ChatGPT. Consider what your expectations are related to the system that is being developed.”

#### Questions about the framework (may omit any if already addressed in previous responses)

1) What would you expect the main goals or outcomes of this system to be?
2) Human oversight refers to the idea that the responses and functioning of the AI system are monitored by humans are monitored by humans rather than having a system that functions completely independently. How much, if any, human monitoring would you expect for this system?
3) What would make this system easy to use?
4) What sort of information would you expect to be given to you about the sources that are used to create this system?
5) How diverse would you expect the data sources used to feed this system to be?
6) What are your expectations regarding user privacy in this system?
7) Different AI systems have different levels of accuracy. This can depend on many factors and can change within the same AI system over time. How would you know if this system is performing well?
8) How well do you expect this system to work?
9) How often would you expect the system to be evaluated for accuracy?
10) What would make this system fair to use with all students?
11) Do you expect the AI system to self-explain its algorithms? If so, what kind of explanations would you need in order to understand the logic of the algorithm?
12) What would you expect to happen if the system receives unexpected questions or questions that don't make sense to it?
13) What would you expect to happen if you [your student] received an answer you disagreed with?
14) Who should be accountable for errors in answers from the system?
15) What sort of impact do you expect this system to have on you and/or your classmates [your students]?
16) Are there any other expectations you would have for this system that haven't been covered by these questions?
17) Do you anticipate that you [your students] would use this kind of system? Why or why not?

#### Discussion of the principles

“I am going to show you a list of principles the designer of an AI system might use when building it. You may have mentioned some of these principles in our discussion already, and others might be things we have not yet talked about. Are there any principles you would expect the designer to use that you have not mentioned already and that are not included in this list?”
